# Impact of Cellular Senescence on LCN2 Expression in Salivary Gland Epithelial Cells and Oral Keratinocytes

**DOI:** 10.1002/biof.70087

**Published:** 2026-02-18

**Authors:** Yosuke Shikama, Kayo Yoshida, Yuka Shikama

**Affiliations:** ^1^ Department of Oral Disease Research National Center for Geriatrics and Gerontology Obu Japan; ^2^ Department of Geriatric Oral Science, Graduate School of Dentistry Tohoku University Sendai Japan

**Keywords:** aging, cellular senescence, inflammaging, LCN2, oral mucosa, saliva, salivary gland

## Abstract

Senescent cells are characterized by the up‐regulation of senescence markers and exhibit key features, such as irreversible growth arrest and the senescence‐associated secretory phenotype (SASP), which is mainly regulated by transcription factors of nuclear factor κB (NF‐κB). Lipocalin‐2 (LCN2), a glycoprotein secreted by immune cells, astrocytes, and epithelial cells, is present in saliva and gingival crevicular fluid and possesses antimicrobial and immunomodulatory properties. Although LCN2 expression is mainly regulated by NF‐κB, the effects of aging and cellular senescence on salivary LCN2 protein concentrations remain unknown. We herein demonstrated that LCN2 protein levels in the serous acinar cells of salivary glands, oral epithelial cells, and saliva were higher in aged mice than in young mice. However, in primary oral keratinocytes and salivary gland epithelial cells, replicative senescence and DNA damage‐induced senescence did not increase LCN2 expression, with similar results being obtained for the SASP factors tumor necrosis factor‐alpha and interleukin‐1β (IL‐1β). Although the cyclic GMP‐AMP synthase‐mediated induction of LCN2 expression has been reported in astrocytes, its expression decreased with cellular senescence, and its ligand did not induce LCN2 expression in these oral‐related epithelial cells. Conversely, an IL‐1β treatment significantly induced LCN2 expression and secretion, even in senescent epithelial cells. The source of IL‐1β was not senescent fibroblasts, but M1 macrophages that accumulate with inflammaging. Collectively, these results suggest that aging up‐regulates LCN2 expression in oral‐related epithelial cells mainly via IL‐1β secreted from M1 macrophages, rather than through the induction of their senescence.

## Graphical abstract

The age‐related increase in salivary LCN2 levels is unlikely to be directly attributable to cellular senescence of oral mucosal or salivary gland epithelial cells, nor to SASP factors derived from surrounding senescent fibroblasts. Instead, it is more plausibly driven by IL‐1β secreted from M1 macrophages that accumulate with inflammaging.

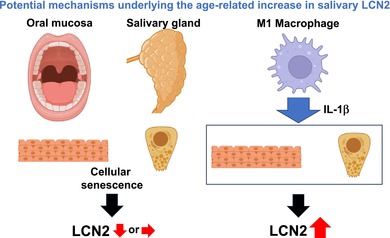

## 1 Introduction

Cellular senescence is a stress response induced by a wide range of intrinsic and extrinsic insults, such as replicative and oxidative stress, non‐telomeric DNA damage, mitochondrial dysfunction, and irradiation [1]. Senescent cells show non‐proliferative irreversible growth arrest, resistance to apoptosis, and marked changes in gene expression, which include dysregulated metabolism and the senescence‐associated secretory phenotype (SASP), as well as the up‐regulation of cyclin‐dependent kinase (CDK) inhibitors, including the CDK2 inhibitor p21^Waf1/Cip1^, the CDK4 and CDK6 inhibitor p16^INK4A^, and transcription factors of nuclear factor κB (NF‐κB) [2]. Although senescence is a potent anticancer mechanism that prevents malignancies by limiting the replication of preneoplastic cells [3], senescent SASP factors and inflammatory mediators from accumulated M1‐polarized macrophages are both key contributors to inflammaging, which is characterized by persistent chronic inflammation and has emerged as a critical factor linked to age‐related diseases [4].

Lipocalin 2 (LCN2), also known as neutrophil gelatinase‐associated lipocalin (NGAL), is a secreted glycoprotein that is produced by immune cells [5], astrocytes [6], and epithelial cells [7]. LCN2 levels are elevated in inflammatory and metabolic disorders, including type 2 diabetes, obesity, kidney disorders, and psoriasis [5]. It was first discovered as a protein that possesses antibacterial properties via iron depletion by binding to bacterial siderophores [8]. However, recent studies reported that LCN2 has multiple functions, including the modulation of inflammatory [9] and anti‐inflammatory responses [10, 11] and chemotaxis [12]. In the oral region, the LCN2 protein exists in saliva [13] and inhibits the adhesion of *Porphyromonas gingivalis* (*Pg*: a major periodontal pathogen) to oral epithelial cells [14]. Moreover, its concentrations in saliva, serum, and gingival crevicular fluid [15, 16, 17] were found to be elevated in patients with periodontitis, suggesting that inflammatory responses induced by bacteria trigger its secretion. LCN2 concentrations have also been shown to increase with age in humans and murine serum [18], indicating that cellular senescence and inflammaging induce LCN2 expression. However, the impact of cellular senescence and aging on LCN2 expression in salivary glands and oral mucosa, which may be factors affecting its protein concentration in saliva, remains unknown.

Therefore, the present study investigated whether aging modulates LCN2 expression in the murine oral mucosa and salivary glands and also the concentration of LCN2 in saliva. The results obtained revealed that LCN2 protein levels on the basal side of the serous acinar cells of salivary glands, the basal layers of oral epithelial cells, and saliva were higher in aged mice than in young mice. However, in primary oral keratinocytes and salivary gland epithelial cells, replicative senescence and DNA damage‐induced senescence did not increase LCN2 expression, and similar results were obtained for interleukin (IL)‐1β and tumor necrosis factor‐alpha (TNF‐α), which are SASP factors [19]. Although a recent study demonstrated that cyclic GMP‐AMP synthase (cGAS) signaling, which senses cytosolic DNA derived from viral/bacterial infection or self‐DNA [20] and drives inflammaging [21], induced LCN2 expression in astrocytes [6], cGAS expression levels significantly decreased with senescence, and signaling did not induce LCN2 expression in these oral‐related cells. Treatment with IL‐1β and TNF‐α, which are also known as proinflammatory cytokines secreted from M1 macrophages [22], significantly increased LCN2 secretion, and IL‐1β induced LCN2 expression even in senescent epithelial cells. Unexpectedly, IL‐1β secretion in gingival fibroblasts, a potential source of IL‐1β in the oral mucosa, did not increase regardless of whether cells were senescent or stimulated with a cGAS agonist. However, stimulation with the conditioned medium (CM: freshly collected after differentiating THP‐1 cells to M1‐like macrophages) increased LCN2 secretion in primary oral keratinocytes, and this increase was significantly inhibited by the addition of an IL‐1 receptor antagonist (IL‐1Ra). These results suggest that aging up‐regulated the expression of LCN2 in salivary gland epithelial cells and oral keratinocytes not via soluble factors secreted by senescent fibroblasts, but mainly via IL‐1β secreted by M1 macrophages that accumulated by inflammaging, rather than through the induction of their senescence.

## 2 Materials and Methods

### 2.1 Reagents and Antibodies

All reagents used in the present study were commercially available, including doxorubicin hydrochloride (DXR) (Fujifilm Wako, Saitama, Japan), G3‐YSD Control (#tlrl‐ydnac, InvivoGen, San Diego, CA, USA), G3‐YSD (#tlrl‐ydna, InvivoGen), Lipofectamine 3000 Reagent (#L3000008, Thermo Fisher Scientific, Waltham, MA, USA), LPS‐PG Ultrapure (#tlrl‐ppglps, InvivoGen), recombinant human IL‐1β protein (#54059, Cell Signaling Technology, Danvers, MA, USA), recombinant human IL‐6 protein (#570802, BioLegend, San Diego, CA, USA), recombinant human TNF‐α protein (#570102, BioLegend), and mouse recombinant IL‐1β protein (#92344, Cell Signaling Technology).

### 2.2 Mice and Saliva Collection

All animal experiments were approved by and conducted in accordance with the guidelines established by the National Center for Geriatrics and Gerontology Animal Ethics Committee (4–19, 3/3/2022). The animal protocol was performed by experienced researchers in accordance with the ARRIVE 2.0 guidelines. Young (age: 4–6 weeks) and aged adult C57BL/6N male mice (age: 18–22 months) were obtained from Japan SLC Inc. (Hamamatsu, Japan) and the Experimental Animal Facility at the National Center for Geriatrics and Gerontology (Obu, Japan), respectively. Mice were housed in specific pathogen‐free conditions under a 12‐h light–dark photocycle and had *ad libitum* access to water and food. Plastic cages of an internal area of 384 cm^2^ × depth of 14 cm with paper bedding were used to house up to a maximum of five animals per cage. The temperature in the room was maintained at 23°C ± 2°C and humidity at 50% ± 10%.

Saliva stimulated by pilocarpine was collected as previously described [23]. Collected samples were immediately cooled on ice and then centrifuged at 3000 × *g* at 4°C for 5 min. The supernatant was frozen at −80°C until analyzed.

### 2.3 RNA Isolation and a Quantitative Real‐Time PCR (qRT‐PCR) Analysis

Total RNA was extracted from cells using the RNeasy mini kit (#74106, Qiagen, Hilden, Germany) according to the manufacturer's instructions. Total RNA concentrations were measured using a Nanodrop spectrophotometer (Thermo Fisher Scientific), and cDNA was synthesized with the PrimeScript RT Master Mix (#RR036A, TaKaRa Bio Inc., Shiga, Japan). qRT‐PCR was performed (amplification for 40 cycles) on a LightCycler 96 system using FastStart Essential DNA Green Master (#06402712001, Roche Applied Science, Mannheim, Germany). The following primers were used for the amplification of specific genes: mouse *Cdkn1a*, 5′‐TCCACAGCGATATCCAGACA‐3′ (sense) and 5′‐GGACATCACCAGGATTGGAC‐3′ (antisense), mouse *Cdkn2b*, 5′‐AATAACTTCCTACGCATTTTCTGC‐3′ (sense) and 5′‐CCCTTGGCTTCAAGGTGAG‐3′ (antisense), mouse *Gapdh*, 5′‐GCCTTCCGTGTTCCTACCC‐3′ (sense) and 5′‐TGAAGTCGCAGGAGACAACC‐3′ (antisense), mouse *Il1b*, 5′‐GCAACTGTTCCTGAACTCAACT‐3′ (sense) and 5′‐ATCTTTTGGGGTCCGTCAACT‐3′ (antisense), mouse *Lcn2*, 5′‐CCCCATCTCTGCTCACTGTC‐3′ (sense) and 5′‐TTTTTCTGGACCGCATTG‐3′ (antisense), mouse *Ccl2* (*monocyte chemoattractant protein‐1: Mcp‐1*), 5′‐TTAAAAACCTGGATCGGAACCAA‐3′ (sense) and 5′‐GCATTAGCTTCAGATTTACGGGT‐3′ (antisense), mouse *Tnf*, 5′‐CTGTAGCCCACGTCGTAGC‐3′ (sense) and 5′‐TTGAGATCCATGCCGTTG‐3′ (antisense), human *CDKN1A*, 5′‐TCACTGTCTTGTACCCTTGTGC‐3′ (sense) and 5′‐GGCGTTTGGAGTGGTAGAAA‐3′ (antisense), human *CDKN2A*, 5′‐GTGGACCTGGCTGAGGAG‐3′ (sense) and 5′‐CTTTCAATCGGGGATGTCTG‐3′ (antisense), human *GAPDH*, 5′‐CATGAGAAGTATGACAACAGCCT‐3′ (sense) and 5′‐AGTCCTTCCACGATACCAAAGT‐3′ (antisense), and human *LCN2*, 5′‐CCACCTCAGACCTGATCCCA‐3′ (sense) and 5′‐CCCCTGGAATTGGTTGTCCTG‐3′ (antisense). The relative quantification of gene expression was performed according to the 2^–ΔΔCT^ method and normalized against *Gapdh* or *GAPDH* mRNA.

### 2.4 Cell Culture and Stimulation

The A‐253 human submandibular gland carcinoma cell line (HTB‐41) and THP‐1 human acute monocytic leukemia cell line (TIB‐202) were purchased from the American Type Culture Collection and cultured in McCoy's 5A (Modified) Medium (#16600082, Thermo Fisher Scientific) or RPMI‐1640 Medium (#R8758, Sigma‐Aldrich, St. Louis, MO, USA), respectively, supplemented with 10% FBS, 100 U/mL penicillin, and 100 μg/mL streptomycin. Human oral keratinocytes (HOK) (#2610) isolated from the oral mucosa (the donor was a 19‐week gestation female) were purchased from ScienCell Research Laboratories (San Diego, CA) and cultured in Defined Keratinocyte‐SFM (#10744019, Gibco, Carlsbad, CA, USA). Human gingival fibroblasts (HGF) (a 22‐year‐old donor) (#P10866, Innoprot, Biscay, Spain) were cultured in Fibroblast Medium (#P60108, Innoprot). The epithelial cells of murine salivary glands (MSEC) were isolated and cultured, and replicative senescence was induced as previously described [24]. When inducing replicative senescence, the number of seeded cells was kept constant, and the number of harvested cells was measured to calculate population doublings. The immortalized human small salivary gland epithelial cell line SV40 (IHSGEC) (#T9289) was purchased from Applied Biological Materials Inc. (BC, V6V 2J5, Canada) and cultured in Defined Keratinocyte‐SFM (Gibco) and Collagen Type I‐coated Dish 100‐mm (#4020‐010, AGC TETHNO GLASS CO. LTD., Shizuoka, Japan). In the replicative senescence experiment, cells were seeded at a density of 1 × 10^6^ cells per well in six‐well plates (to collect lysates) or 5 × 10^5^ cells per well in 12‐well plates (to collect total RNA), and samples were collected when cells reached 90% confluency. In DXR‐induced senescence experiments, cells were seeded at a density of 1 × 10^6^ cells per well in six‐well plates (to collect lysates) or 5 × 10^5^ cells per well in 12‐well plates (to collect total RNA) and incubated overnight. After cells were treated with DXR at the dose described in the figure legends for 48 h, the supernatants were removed and cells were cultured in medium alone for 24 h before sampling [25]. In experiments stimulated with a cGAS agonist (G3‐YSD), recombinant IL‐1β, TNF‐α, or IL‐6, cells were seeded at a density of 1 × 10^6^ cells per well in six‐well plates (to collect lysates) or 5 × 10^5^ cells per well in 12‐well plates (to collect total RNA or supernatants) and then incubated overnight. Cells were treated with these stimulants at the doses described in the figure legends for 24 or 48 h before sampling. G3‐YSD Control and G3‐YSD were transfected with Lipofectamine 3000 transfection reagent according to the manufacturer's instructions.

### 2.5 Collection of CM From THP‐1‐Derived M1 Macrophages

THP‐1 monocytes were differentiated into M1‐like macrophages as previously described [26]. Briefly, 2 × 10^6^ THP‐1 monocytes in 6‐cm dishes were differentiated into macrophages by a 24‐h incubation with 150 nM phorbol 12‐myristate 13‐acetate (#162‐23591, Fujifilm Wako) followed by a 24‐h incubation in 10% FBS RPMI medium. After macrophages were polarized into M1 macrophages by a 24‐h incubation with 20 ng/mL of recombinant human IFN‐γ protein (#570204, BioLegend) and 10 pg/mL of *Escherichia coli* lipopolysaccharide (LPS) (#tlrl‐eblps, InvivoGen), the medium was removed, cells were washed with PBS, and were then incubated in 10% FBS RPMI medium for 24 h to avoid contamination of the CM with IFN‐γ and LPS. The CM was collected and centrifuged at 500 × *g* to remove cellular debris, diluted twice with Defined Keratinocyte‐SFM, and then transferred to monolayers of HOK in a 12‐well plate. Recombinant human IL‐1Ra protein (#280‐RA‐010, R&D Systems, Minneapolis, MN, USA) was added just before the diluted CM was transferred to HOK.

### 2.6 Western Blot Analysis

After the treatment, cells were washed twice with ice‐cold PBS and lysed in RIPA buffer supplemented with a 1% protease and phosphatase inhibitor cocktail (#78440, Thermo Fisher Scientific). Lysates were centrifuged at 12,000 × *g* at 4°C for 10 min and the supernatants were collected. The protein concentrations of lysates were assessed using the BCA protein assay kit (#23227, Thermo Fisher Scientific). Protein concentrations were adjusted, and samples were then diluted in 2× or 4× Laemmli Sample Buffer. After boiling at 95°C for 5 min, proteins were separated using sodium dodecyl sulfate‐polyacrylamide gel electrophoresis and transferred to polyvinylidene difluoride membranes (#1620175, Bio‐Rad, Hercules, CA, USA). The following primary antibodies were used: α‐amylase (1:4000, #A8273, Sigma‐Aldrich), ADFP (Perilipin 2) (1:250, #PA1‐16972, Thermo Fisher Scientific), aquaporin 5 (AQP5) (1:400, #AQP‐005, Alomone Labs, Jerusalem, Israel), cGAS (1:1000, for murine cell lysates; #31659, for human cell lysates; #79978, Cell Signaling Technology), cytokeratin 19 (1:2000, #MABT913, Sigma‐Aldrich), GAPDH (1:5000, #5174, Cell Signaling Technology), IκBα (1:1000, #4812, Cell Signaling Technology), cytokeratin 5 (1:2000, #ab52635, Abcam, Cambridge, MA, USA), Lamin B1 (1:1000, #17416, Cell Signaling Technology), NGAL (1:200, for murine cell lysates, #sc‐515876, Santa Cruz Biotechnology, CA, USA), LCN2 (1:1000, for human cell lysates, #44058, Cell Signaling Technology), NF‐κB p65 (1:1000, #8242, Cell Signaling Technology), p16 INK4A (1:1000, #29271, Cell Signaling Technology), p21 Waf1/Cip1 (1:1000, #2947, Cell Signaling Technology), phospho‐Histone H2A.X (1:1000, #9718, Cell Signaling Technology), and phospho‐NF‐κB p65 (1:1000, #3033, Cell Signaling Technology). Proteins were visualized with Immunostar (Fujifilm Wako) and Amersham Imager 680, and the optical densities of protein bands were measured with Amersham Imager 680 Analysis Software (GE Healthcare, Piscataway, NJ, USA).

### 2.7 Sample Preparation for Immunohistochemical Staining

Archived paraffin‐embedded tissue specimens were used. Tissue sections (thickness of 4 μm) were deparaffinized in xylene and rehydrated in descending grades of ethanol. Antigen retrieval was performed using a pressure cooker and citrate phosphate buffer (pH 6.0). Endogenous peroxidase activity was blocked with 3% H_2_O_2_ for 10 min. Sections were treated with the following antibodies: Normal Rat IgG (1:500, #147‐09521, Fujifilm Wako), Normal Mouse IgG (1:5000, #140‐09511, Fujifilm Wako), Lipocalin‐2/NGAL (1:50, #ab70287, Abcam), or NGAL (1:100, # sc‐515876, Santa Cruz Biotechnology). After sections were incubated at 4°C overnight, they were washed with PBS and treated with the ImmPRESS HRP Goat Anti‐Rat IgG (Mouse Adsorbed) Polymer Kit (#MP‐7444, Vector Laboratories, Newark, CA, USA) or M.O.M ImmPRESS Peroxidase Polymer Kit (#MP‐2400, Vector Laboratories). The reaction was detected by diaminobenzidine staining (#MK210, TaKaRa). Sections were then counterstained with hematoxylin, dehydrated in ascending grades of ethanol, and mounted on slides.

### 2.8 Enzyme‐Linked Immunosorbent Assay (ELISA)

MCP‐1 and LCN2 concentrations in saliva and supernatants collected from MSEC were measured using the OptEIA Mouse MCP‐1 ELISA set (#555260, BD Biosciences, San Diego, CA, USA) or Mouse Lipocalin‐2/NGAL ELISA Kit (MLCN20, R&D Systems), respectively. IL‐1β, LCN2, and TNF‐α concentrations in supernatants collected from HGF, HOK, and IHSGEC were measured using the ELISA MAX Deluxe Set Human IL‐1β (#437004, BioLegend), Human Lipocalin‐2/NGAL DuoSet ELISA (#DY1757, R&D Systems), and Human TNF‐alpha DuoSet ELISA (#DY210‐05, R&D Systems), respectively. Optical density at 450 nm was measured using a microplate reader (#51119050, Thermo Fisher Scientific).

### 2.9 Senescence‐Associated β‐Galactosidase (SA‐βGal) Activity

After reaching 90% confluency in a collagen type I‐coated 60‐mm dish (#4010–010, AGC TETHNO GLASS CO. LTD.), MSEC were detached with TrypLE Express Enzyme (#12604021, Thermo Fisher Scientific) and stained with SPiDER‐βGal (#SG02, DOJINDO LABORATORIES, Kumamoto, Japan). Expression was detected using a Canto II flow cytometer and analyzed with FlowJo software (BD Biosciences).

### 2.10 Data and Statistical Analyses

Data from the tissues and saliva of aged mice with obvious malignant tumors were excluded. Investigators were not blinded during data collection. Based on our previous studies, the sample size in the present study was adequately powered to detect the observed differences between the experimental groups. The declared group size was the number of independent values, and statistical analyses were performed using these values. The significance of differences was examined using the Student's unpaired *t*‐test or Dunnett's multiple comparison test after analyzing variance using GraphPad Prism (version 10.0.1 GraphPad Software). *p*‐values < 0.05 were considered to be significant.

## 3 Results

### 3.1 Effects of Aging on LCN2 Expression in Murine Salivary Glands and Oral Mucosa and on Salivary LCN2 Concentrations

**FIGURE 1:**
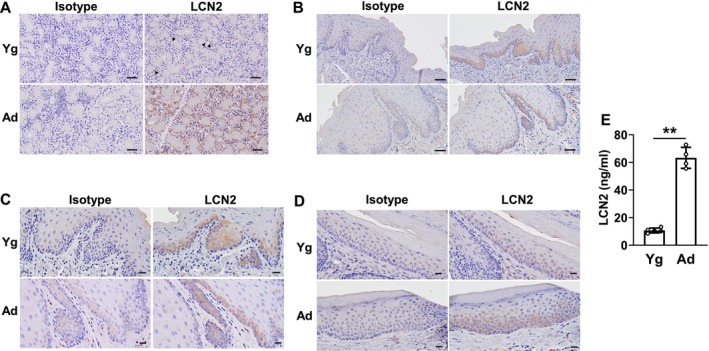
Aging affects LCN2 expression in murine submandibular glands and oral mucosa and salivary LCN2 concentrations. (A) Sections of submandibular glands from young (Yg) and aged (Ad) mice were stained with an isotype or anti‐LCN2 antibody and counterstained with hematoxylin. Arrowheads show the positive staining area of LCN2 in Yg mice. (B–D) Sections of the masticatory mucosa (B and C) and lining mucosa (D) from Yg and Ad mice were stained with the isotype or anti‐LCN2 antibody and counterstained with hematoxylin. Higher magnification pictures are shown in (C). Representative pictures of 3 mice each. Bar: 50 μm (A and B) and 20 μm (C and D). (E) Detection of LCN2 by ELISA in saliva collected from Yg (*N* = 5) and Ad (*N* = 4) mice. Data represent the mean ± standard deviation (SD). **p < 0.01 (the unpaired Student's *t*‐test).

We investigated the localization of LCN2 expression in murine salivary glands and the oral epithelium and whether aging affected its expression. The expression of senescence markers was previously shown to increase in both the salivary glands [23] and oral mucosa [27] with aging. In the salivary glands of young mice, the low expression of LCN2 was observed on the basal side of serous acinar cells. The level and area of expression were both markedly increased in aged mice (Figure 1A). In the masticatory mucosa, LCN2 expression was detected in the basal layer of the epithelium regardless of age (Figure 1B,C). Staining of the basal layer of the lining mucosa was stronger in aged mice than in young mice (Figure 1D). Moreover, the concentration of LCN2 in saliva was significantly higher in aged mice than in young mice (Figure 1E). These results suggest that aging increased the salivary concentration of LCN2, which may be attributed to its up‐regulated expression in the salivary glands and oral mucosa.

### 3.2 Replicative Senescence Decreases LCN2 Expression in MSEC and HOK

**FIGURE 2:**
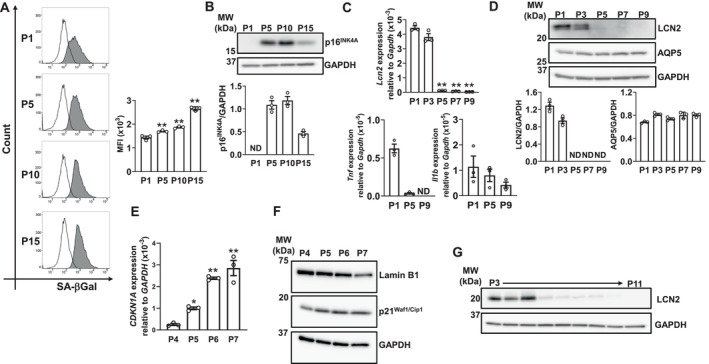
Effects of replicative senescence on LCN2 expression in MSEC and HOK. The number following “P” indicates the passage number. (A) Detection of SA‐βGal in MSEC by flow cytometry. The gray area indicates the sample treated with the reagent, while the black line represents the untreated control. A representative histogram from three independent experiments is shown. The bar graph shows the mean fluorescence intensity (MFI) of each experiment. Data represent the mean ± standard error of the mean (SEM) of triplicate assays. **p < 0.01 versus P1 cells (Dunnett's multiple comparison test). (B) Whole‐cell lysates were prepared from MSEC passaged as described. Cell lysates were immunoblotted with anti‐p16^INK4A^ and anti‐GAPDH antibodies. A representative blot of three independent experiments is shown. The bar graph shows the integrated signal intensities of the p16^INK4A^/GAPDH ratio and the mean ± SEM of triplicate assays. ND: Not detected. (C) *Lcn2*, *Tnf*, and *Il1b* mRNA expression levels in MSEC were quantified using real‐time PCR. Data represent the mean ± SEM of triplicate assays. ND, not detected. **p < 0.01 versus P1 cells (Dunnett's multiple comparison test). (D) Whole‐cell lysates were prepared from MSEC passaged as described. Cell lysates were immunoblotted with anti‐LCN2, anti‐AQP5, and anti‐GAPDH antibodies. A representative blot of three independent experiments is provided. The bar graph shows the integrated signal intensities of the LCN2/GAPDH or AQP5/GAPDH ratio and the mean ± SEM of triplicate assays. ND: Not detected. (E) *CDKN1A* mRNA expression levels in HOK passaged as described were quantified using real‐time PCR. Data represent the mean ± SEM of triplicate assays. *p < 0.05 and **p < 0.01 versus P4 cells (Dunnett's multiple comparison test). (F) Whole‐cell lysates were prepared from HOK passaged as described. Cell lysates were immunoblotted with anti‐Lamin B1, anti‐p21^Waf1/Cip1^, and anti‐GAPDH antibodies. A representative blot of three independent experiments is shown. The bar graph shows the integrated signal intensity ratios of Lamin B1/GAPDH and p21^Waf1/Cip1^/GAPDH, expressed as the mean ± SEM from triplicate assays, in Figure S2. (G) Whole‐cell lysates were prepared from HOK passaged as described. Cell lysates were immunoblotted with anti‐LCN2 and anti‐GAPDH antibodies. A representative blot of two independent experiments is shown.

We previously established a replicative senescence model in MSEC [24], and examined the effects of replicative senescence on LCN2 expression in this model. Increases in the expression of the senescence markers, SA‐βGal and P16^INK4A^, were confirmed with successive passages by flow cytometry (Figure 2A) and Western blotting (Figure 2B), respectively. We also confirmed that the expression of perilipin 2, a protein indicated to accumulate lipids in senescence [28], was significantly increased by repeated passages in MSCE (Figure S1). A previous study demonstrated that LCN2 expression was regulated by NF‐κB as well as IL‐1β and TNF‐α [7]. LCN2 gene expression markedly decreased after five passages and did not recover through at least nine passages. The gene expression of IL‐1β and TNF‐α, which are SASP factors, also decreased with continued passaging; however, their expression levels were markedly lower than that of LCN2 (Figure 2C). Moreover, the level of the LCN2 protein became undetectable after five passages, whereas the level of the acinar marker AQP5 remained unchanged (Figure 2D), suggesting that the reduction in LCN2 expression was not caused by a decrease in the number of acinar cells with passaging, but by the down‐regulated expression of LCN2 in acinar cells. In HOK, increases in senescence markers [28] with passaging were confirmed by real‐time PCR (Figure 2E) and by Western blotting (Figure 2F and Figure S2), while marked decreases were observed in LCN2 protein expression (Figure 2G). These results indicate that replicative senescence down‐regulated LCN2 expression in these cells.

### 3.3 Effects of DNA Damage‐Induced Senescence on LCN2 Expression

**FIGURE 3:**
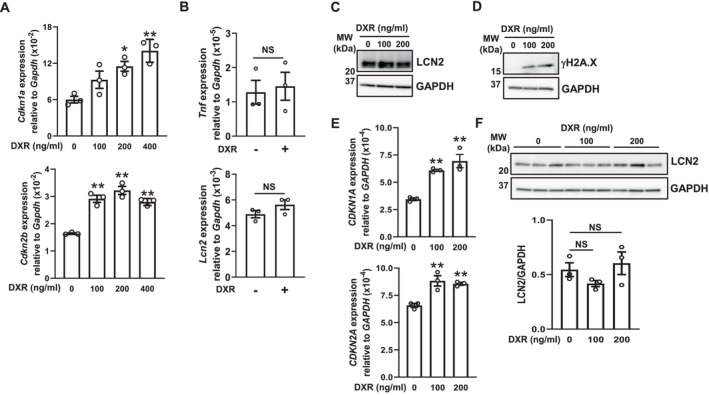
Effects of DXR‐induced senescence on LCN2 expression in MSEC and HOK. These experiments were conducted using MSEC or HOK passaged three times. (A) MSEC were treated with the indicated concentrations of DXR as described in the Materials and Methods. *Cdkn1a* and *Cdkn2b* mRNA expression levels were quantified using real‐time PCR. Data represent the mean ± SEM of triplicate assays. *p < 0.05 and **p < 0.01 versus the DXR 0 ng/mL group (Dunnett's multiple comparison test). (B) MSEC were treated with (+) or without (−) DXR (200 ng/mL) as described in the Materials and Methods. *Tnf* and *Lcn2* mRNA expression levels were quantified using real‐time PCR. Data represent the mean ± SEM of triplicate assays. NS, not significant. (the unpaired Student's *t*‐test). (C) MSEC were treated with the indicated concentrations of DXR as described in the Materials and Methods. Whole‐cell lysates were immunoblotted with anti‐LCN2 and anti‐GAPDH antibodies. A representative blot of two independent experiments is shown. (D) HOK were treated with the indicated concentrations of DXR as described in the Materials and Methods. Whole‐cell lysates were immunoblotted with anti‐γH2A.X and anti‐GAPDH antibodies. A representative blot of two independent experiments is shown. (E) HOK were treated with the indicated concentrations of DXR as described in the Materials and Methods. *CDKN1A* and *CDKN2A* mRNA expression levels were quantified using real‐time PCR. Data represent the mean ± SEM of triplicate assays. **p < 0.01 versus the DXR 0 ng/mL group (Dunnett's multiple comparison test). (F) HOK were treated with the indicated concentrations (*N* = 3) of DXR as described in the Materials and Methods. Whole‐cell lysates were immunoblotted with anti‐LCN2 and anti‐GAPDH antibodies. A representative blot of two independent experiments is shown. The bar graph shows the integrated signal intensities of the LCN2/GAPDH ratio and the mean ± SEM of triplicate assays. NS, not significant (Dunnett's multiple comparison test).

We investigated the effects of DNA damage‐mediated cellular senescence by DXR [25, 29] on LCN2 expression. Although the DXR treatment increased senescence markers related to CDK inhibitors (Figure 3A), the mRNA expression levels of LCN2 and TNF‐α (Figure 3B) as well as the protein level of LCN2 (Figure 3C) remained unchanged in MSEC. In HOK, the DXR treatment increased γH2A.X (Figure 3D), a marker of DNA double‐strand breaks that is frequently present in senescent cells [25], and the mRNA expression levels of CDK inhibitors (Figure 3E), whereas the LCN2 protein expression level remained unchanged (Figure 3F). These results suggest that DNA damage‐mediated cellular senescence also did not induce LCN2 expression.

### 3.4 Effects of Cellular Senescence on cGAS Expression and the Potential Induction of LCN2 Expression by cGAS Signaling

**FIGURE 4:**
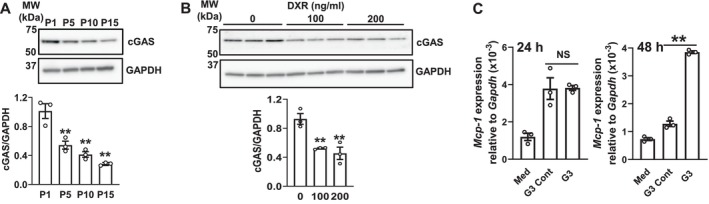
Effects of cellular senescence on cGAS expression and of G3‐YSD on MCP‐1 mRNA expression. The number following “P” indicates the passage number. (A) Whole‐cell lysates were prepared from MSEC passaged as described. Cell lysates were immunoblotted with anti‐cGAS and anti‐GAPDH antibodies. A representative blot of three independent experiments is shown. The bar graph shows the integrated signal intensities of the cGAS/GAPDH ratio and the mean ± SEM of triplicate assays. **p < 0.01 versus P1 cells (Dunnett's multiple comparison test). (B) HOK (P3) were treated with the indicated concentrations (*N* = 3) of DXR as described in the Materials and Methods. Whole‐cell lysates were immunoblotted with anti‐cGAS and anti‐GAPDH antibodies. A representative blot of two independent experiments is shown. The bar graph shows the integrated signal intensities of the cGAS/GAPDH ratio and the mean ± SEM of triplicate assays. **p < 0.01 versus the DXR 0 ng/mL group (Dunnett's multiple comparison test). (C) MSEC (P3) were treated with medium alone (Med), G3‐YSD Control (G3 Cont), or G3‐YSD (G3) (1 μg/mL each) as described in the Materials and Methods for 24 or 48 h. *Mcp‐1* mRNA expression levels were quantified using real‐time PCR. Data represent the mean ± SEM of triplicate assays. NS, not significant. **p < 0.01 (the unpaired Student's *t*‐test).

**FIGURE 5:**
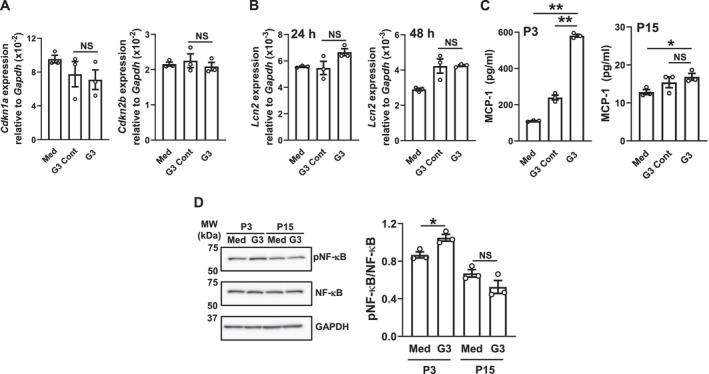
Effects of cGAS signaling on cellular senescence and LCN2 mRNA expression, and the involvement of cellular senescence in MCP‐1 secretion. The number following “P” indicates the passage number. (A) MSEC (P3) were treated with medium alone (Med), G3‐YSD Control (G3 Cont), or G3‐YSD (G3) (1 μg/mL each) for 48 h. *Cdkn1a* and *Cdkn2b* mRNA expression levels were quantified using real‐time PCR. Data represent the mean ± SEM of triplicate assays. NS, not significant. (the unpaired Student's *t*‐test). (B) MSEC (P3) were treated with Med, G3 Cont, or G3 (1 μg/mL each) for 24 or 48 h. *LCN2* mRNA expression levels were quantified using real‐time PCR. Data represent the mean ± SEM of triplicate assays. NS, not significant (the unpaired Student's *t*‐test). (C) MSEC, P3 or P15, were treated with Med, G3 Cont, or G3 (1 μg/mL each) for 48 h. The concentration of MCP‐1 in the medium was assayed by ELISA. Data represent the mean ± SEM of triplicate assays. NS, not significant. *p < 0.05 and **p < 0.01 (the unpaired Student's *t*‐test). (D) MSEC, P3 or P15, were treated with Med or G3 (1 μg/mL) for 3 h. Cell lysates were immunoblotted with anti‐phospho‐(p)NF‐κB, anti‐NF‐κB, and anti‐GAPDH antibodies. A representative blot of three independent experiments is shown. The bar graph shows the integrated signal intensities of the pNF‐κB/NF‐κB ratio and the mean ± SEM of triplicate assays. NS, not significant. *p < 0.05 (the unpaired Student's *t*‐test).

Recent studies demonstrated that abnormal cytoplasmic DNA fragments produced during cellular senescence acted as ligands of the DNA sensor, cGAS‐stimulator of interferon genes (STING), and induced the expression of a series of SASP factors [30, 31] and LCN2 [6]. Therefore, we examined the effects of cellular senescence on cGAS expression in these primary cells. Replicative and DNA‐damaged senescence significantly decreased cGAS expression in MSEC (Figure 4A) and HOK (Figure 4B), respectively. A 48‐h treatment with G3‐YSD, a cGAS agonist, significantly increased the mRNA expression of MCP‐1 (Figure 4C), but not senescence markers (Figure 5A) or LCN2 (Figure 5B) in MSEC. Similar to IL‐1β and TNF‐α, MCP‐1 expression is regulated by the NF‐κB signaling pathway [32]. The G3‐YSD treatment also induced MCP‐1 secretion in MSEC after three passages; however, its induction was negligible after 15 passages (Figure 5C). Moreover, IκBα degradation (Figure S3) and NF‐κB activation (Figure 5D) induced by the G3‐YSD treatment were confirmed in MSEC after three passages, but not after 15 passages. NF‐κB is activated through the phosphorylation of IκBα, which is followed by the proteasome‐mediated degradation of IκBα [33]. These results suggest that cellular senescence decreased cGAS expression and its mediated signaling, and also that cGAS signaling did not induce cellular senescence or LCN2 expression in these cells.

### 3.5 Candidate Factors Inducing LCN2 Expression in Oral and Salivary Gland Epithelial Cells

**FIGURE 6:**
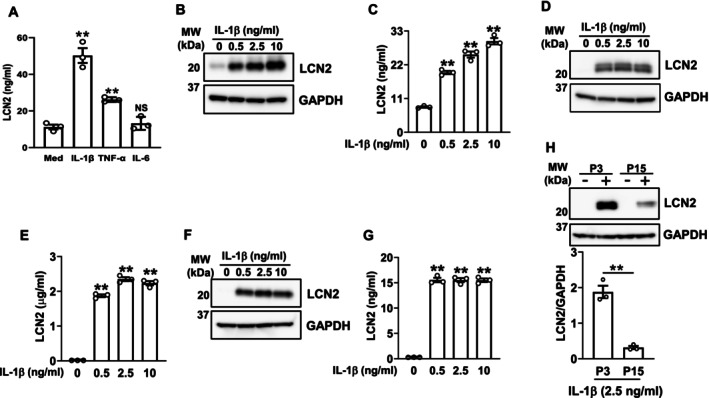
IL‐1β stimulates LCN2 expression in HOK, MSEC, and IHSGEC. In experiments using HOK or MSEC, cells passaged three times were used, unless otherwise indicated. (A) HOK were treated with IL‐1β (500 pg/mL), TNF‐α (20 ng/mL), or IL‐6 (50 ng/mL) as described in the Materials and Methods for 48 h. The concentration of LCN2 in the medium was assayed by ELISA. Data represent the mean ± SEM of triplicate assays. NS, not significant. **p < 0.01 versus medium alone (Med) (the unpaired Student's *t*‐test). (B, C) HOK were treated with the indicated concentrations of IL‐1β as described in the Materials and Methods for 48 h. (B) Whole‐cell lysates were immunoblotted with anti‐LCN2 and anti‐GAPDH antibodies. A representative blot of two independent experiments is shown. (C) The concentration of LCN2 in the medium was assayed by ELISA. Data represent the mean ± SEM of triplicate assays. **p < 0.01 versus the IL‐1β 0 ng/mL group (Dunnett's multiple comparison test). (D, E) MSEC were treated with the indicated concentrations of IL‐1β as described in the Materials and Methods for 48 h. (D) Whole‐cell lysates were immunoblotted with anti‐LCN2 and anti‐GAPDH antibodies. A representative blot of two independent experiments is shown. (E) The concentration of LCN2 in the medium was assayed by ELISA. Data represent the mean ± SEM of triplicate assays. **p < 0.01 versus the IL‐1β 0 ng/mL group (Dunnett's multiple comparison test). (F, G) IHSGEC were treated with the indicated concentrations of IL‐1β as described in the Materials and Methods for 48 h. (F) Whole‐cell lysates were immunoblotted with anti‐LCN2 and anti‐GAPDH antibodies. A representative blot of two independent experiments is shown. (G) The concentration of LCN2 in the medium was assayed by ELISA. Data represent the mean ± SEM of triplicate assays. **p < 0.01 versus the IL‐1β 0 ng/mL group (Dunnett's multiple comparison test). (H) MSEC, passaged three (P3) or 15 times (P15), were treated with (+) or without (−) IL‐1β (2.5 ng/mL) for 48 h. Whole‐cell lysates were immunoblotted with anti‐LCN2 and anti‐GAPDH antibodies. A representative blot of three independent experiments is shown. The bar graph shows the integrated signal intensities of the LCN2/GAPDH ratio and the mean ± SEM of triplicate assays. **p < 0.01 (the unpaired Student's *t*‐test).

Since we previously reported that replicative and oxidative stress‐induced senescence both increased SASP factors, such as IL‐1β and TNF‐α, in gingival fibroblasts [34], and these cytokines have been shown to induce LCN2 expression in epithelial cells [7], we next examined the effects of IL‐1β and TNF‐α on LCN2 production in HOK. A TNF‐α stimulation at 20 ng/mL significantly increased LCN2 production, whereas IL‐1β at 500 pg/mL induced a two‐fold increase in LCN2 production from that with the TNF‐α stimulation. However, IL‐6, also known as a SASP factor [35], did not significantly increase LCN2 production, even at a concentration of 50 ng/mL (Figure 6A). The IL‐1β stimulation dose‐dependently induced LCN2 expression (Figure 6B) and secretion (Figure 6C) in HOK, which were confirmed by Western blotting and ELISA, respectively. Similarly, the IL‐1β stimulation of MSEC induced the intracellular expression (Figure 6D) and secretion (Figure 6E) of LCN2, with the latter reaching high levels in the microgram range. A similar effect was observed in IHSGEC (Figure 6F,G), in which the presence of serous acinar cells was confirmed by Western blotting (Figure S4). The induction of LCN2 expression by IL‐1β was also confirmed in MSEC, even after 15 passages (Figure 6H). These results suggest that IL‐1β derived from neighboring cells induced LCN2 expression in epithelial cells, and also that this induction may even occur in senescent epithelial cells.

### 3.6 Potential Cellular Sources of IL‐1β in the Oral Mucosa During Aging

**FIGURE 7:**
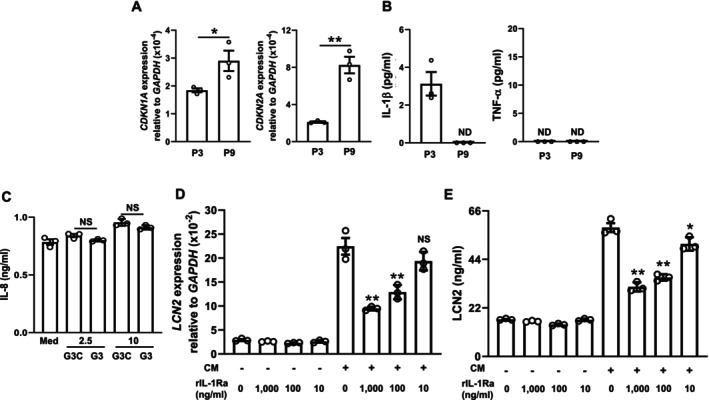
M1 macrophage‐derived IL‐1β induces LCN2 expression in oral epithelial cells. (A) CDKN1A and CDKN2A mRNA expression levels in HGF passaged three times (P3) or nine times (P9) were quantified using real‐time PCR. Data represent the mean ± SEM of triplicate assays. *p < 0.05 and **p < 0.01 (the unpaired Student's *t*‐test). (B) HGF, passaged P3 or P9, were cultured as described in the Materials and Methods for 48 h. The concentration of IL‐1β and TNF‐α in the medium was assayed by ELISA. Data represent the mean ± SEM of triplicate assays. ND, not detected. (C) HGF (P3) were treated with medium alone (Med), G3‐YSD Control (G3C), or G3‐YSD (G3) (2.5 or 10 μg/mL each) for 48 h. The concentration of IL‐8 in the medium was assayed by ELISA. Data represent the mean ± SEM of triplicate assays. NS, not significant. (the unpaired Student's *t*‐test). (D, E) HOK were treated with the conditioned medium as described in the Materials and Methods for 24 h. (D) *LCN2* mRNA expression levels were quantified using real‐time PCR. (E) The concentration of LCN2 in the medium was assayed by ELISA. Data represent the mean ± SEM of triplicate assays. NS: Not significant. *p < 0.05 and **p < 0.01 versus the CM(+) and IL‐1Ra 0 ng/mL group (Dunnett's multiple comparison test).

We initially hypothesized that senescent fibroblasts may be a source of IL‐1β. Although repeated passaging significantly increased the expression of senescent markers in HGF (Figure 7A), the secreted protein levels of IL‐1β and TNF‐α were not increased (Figure 7B), whereas the expression of the corresponding genes was up‐regulated, as previously reported [34]. Furthermore, even upon stimulation with a high concentration of a cGAS agonist, the production of IL‐8 (Figure 7C) and IL‐1β (Figure S5), both of which are transcriptionally regulated by NF‐κB [35], was not increased. We then investigated whether M1 macrophages are a source of IL‐1β because an age‐associated increase in inflammatory M1 macrophages has been documented in various tissues, including neural tissue [36], oral mucosa [37], and salivary glands [38]. To achieve this, HOK were treated with CM collected from M1 macrophages that differentiated from THP‐1. Under this experimental condition, we confirmed that CM markedly induced LCN2 expression at both the mRNA (Figure 7D) and protein (Figure 7E) levels, and this induction was suppressed in a concentration‐dependent manner with IL‐1Ra, which binds to IL‐1 receptor type I without signal transduction, serving as a naturally occurring antagonist [39]. Collectively, these findings indicate that M1 macrophages, rather than senescent fibroblasts, are a likely source of IL‐1β that induce LCN2 production in the oral mucosa during aging.

## 4 Discussion

We herein demonstrated that replicative and DNA damage‐induced senescence did not increase IL‐1β, TNF‐α, or LCN2 expression in MSEC and HOK. A previous study reported that the induction of SASP after DNA damage was independent of p16^INK4a^ expression, suggesting that cell cycle arrest does not necessarily lead to SASP [40]. Moreover, the overexpression of p16^INK4a^ repressed the transactivation of NF‐κB; therefore, p16^INK4a^ is a potent inhibitor of NF‐κB signaling [41]. The effective tumor suppressor, p14^ARF^, which activates the p53/p21^Waf1/Cip1^ pathway, has also been identified as an inhibitor of NF‐κB signaling [42]. Although the composition and mechanisms of SASP induction are generally shared by all cell types [43], specificity regarding epithelial SASP appears to exist based on differences in the expression patterns of SASP factors [44, 45]. We also confirmed a decrease in NF‐κB phosphorylation levels upon repeated passaging (Figure S6). These results suggest that even in epithelial cells, the composition of SASP factors and the associated transcriptional regulators differ among cell types.

SASP factors also act in a paracrine manner, inducing the senescence of surrounding cells, which is termed paracrine senescence [46]. Since we previously demonstrated that cellular senescence in gingival fibroblasts increased the gene expression of SASP factors, such as IL‐1β and TNF‐α [34], we initially suspected senescent fibroblasts as a potential source of these cytokines. However, in the present study, these increases were not observed at the secreted protein level (Figure 7B). Therefore, when investigating paracrine senescence, it is crucial to evaluate protein levels rather than relying solely on mRNA expression levels.

We showed that the IL‐1β treatment significantly increased LCN2 expression and secretion in oral keratinocytes (Figure 6A–C) and salivary gland epithelial cells (Figure 6D–G), even in senescent cells (Figure 6H). In comparisons with NF‐κB activators, such as TNF‐α and LPS, IL‐1 signaling selectively up‐regulated LCN2 expression [7]. We also confirmed that even at a high concentration of 10 μg/mL, *Pg* LPS failed to induce LCN2 expression in HOK (Figure S7). Moreover, IL‐1 signaling has been shown to induce cellular senescence [46], and we herein revealed that the IL‐1β treatment increased γH2A.X expression levels in HOK (Figure S8). Although LCN2 in gingival crevicular fluid and saliva may be a biomarker for periodontitis [17, 47], its production, and potentially its induction of cellular senescence, may be indirectly mediated through IL‐1 signaling or other factors from surrounding cells rather than being directly triggered by the virulence factors of periodontal pathogens in oral epithelial cells.

The immunohistochemical staining results shown in Figure 1B–D demonstrate that LCN2 is mainly expressed in the basal layer of the oral mucosal epithelium; therefore, LCN2 expression may also be involved in epithelial differentiation. LCN2 expression is promoted in keratinocytes upon HPV infection, which confers keratinocytes with the property of poor differentiation [48]. LCN2 is highly expressed in diseases characterized by defective keratinocyte differentiation, such as psoriasis vulgaris, condyloma acuminatum, and squamous cell carcinoma [49]. Since the replicative senescence–associated down‐regulation of LCN2 expression in HOK was also observed for keratin 5 (a basal layer marker) (Figure S9), the degree of keratinocyte differentiation may be an important factor regulating LCN2 expression together with expression pathways by cellular senescence.

LCN2 has a number of biological functions, including antimicrobial activity [8, 14], pro‐inflammatory [9] and anti‐inflammatory [10, 11] effects, and chemotaxis [12], which are dependent on its chemical structure, concentration, duration of exposure, and the presence and absence of receptor expression. While the antimicrobial function of LCN2 has been attributed to its iron‐binding capacity through competition with bacterial siderophores, such as Enterobactin [8], recent evidence suggests that LCN2 also inhibits the adhesion of *Pg* through a siderophore‐independent mechanism. This inhibitory effect may be derived from the essential role of iron in the proliferation and synthesis of structural components in *Pg* [14]. Olson et al. reported different roles for LCN2 in neuronal dysfunction depending on the duration of exposure [50]. Although LCN2 at a high concentration (11 μg/mL) has been shown to increase the expression of pro‐inflammatory cytokines in human neutrophils from patients with psoriasis and induce neutrophil chemotaxis [12], LCN2 (0.5–2 μg/mL) reduced the expression of pro‐inflammatory cytokines in murine retinal cells [51]. LCN2 has three known receptors: megalin (LRP2), 24p3R (SLC22A17), and the type 4 melanocortin receptor (MC4R). While megalin expression has been detected in the oral epithelial tissues of patients with oral lichen planus, leukoplakia, and squamous cell carcinoma, its expression was absent in a healthy oral epithelium [52]. Moreover, 24p3R expression was negligible in cells constituting periodontal tissues [10]. Based on these findings, an increase in salivary LCN2 concentrations may enhance its antimicrobial activity and be beneficial to the host; however, further comprehensive studies are needed.

A limitation of the present study is that we did not evaluate the cytoplasmic accumulation of endogenous nucleic acids that act as cGAS ligands in senescent cells. In vascular endothelial cells, replicative senescence also leads to a reduction in cGAS expression levels; however, this is accompanied by the accumulation of cytosolic double‐stranded DNA (dsDNA), which has been shown to induce the expression of SASP factors, such as IL‐1β, IL‐6, and IL‐8, regulated by NF‐κB [35]. In the present study, the expression of IL‐1β, TNF‐α, and LCN2 did not increase with replicative senescence or DXR‐induced senescence in oral‐related epithelial cells. These results suggest that dsDNA did not accumulate in the cytosol to a sufficient level for the expression of these genes. This discrepancy may be attributable, at least in part, to differences in cell types.

In conclusion, the present study indicates that aging increased salivary LCN2 concentrations, and also that the expression of LCN2 in salivary gland epithelial cells and oral keratinocytes was not directly induced by its cellular senescence or indirectly by soluble factors secreted from senescent fibroblasts, but rather by IL‐1β secreted from M1 macrophages accumulated by inflammaging. These results provide insights into the mechanisms underlying the effects of aging on salivary LCN2 concentrations, and will encourage further studies on enhancements in host antimicrobial activity via the salivary glands and oral mucosa.

## Author Contributions

Yosuke Shikama contributed to the conception and design of the study, data acquisition and interpretation, performed all statistical analyses, and drafted and critically revised the manuscript. Kayo Yoshida contributed to data acquisition and analysis, drafted and critically revised the manuscript. Yuka Shikama contributed to data acquisition, drafted and critically revised the manuscript. All authors gave their final approval and agreed to be accountable for all aspects of the work.

## Funding

The authors disclosed receipts of the following financial support for the research, authorship, and/or publication of this article: this work was supported by Research Funding for Longevity Sciences from the National Center for Geriatrics and Gerontology (#24‐4 to Yosuke Shikama) and JSPS KAKENHI (Grant Number 21H03115 to Yosuke Shikama). The funder had no role in the study design, data collection and analysis, decision to publish, or manuscript preparation.

## Conflicts of Interest

The authors declare no conflicts of interest.

## Supporting information


**Figure S1:** The number following “P” indicates the passage number. Whole‐cell lysates were prepared from MSEC passaged as described. Cell lysates were immunoblotted with anti‐Perilipin 2 and anti‐GAPDH antibodies. The bar graph shows the integrated signal intensities of the Perilipin 2/GAPDH ratio and the mean ± SEM of triplicate assays. *p < 0.05 (the unpaired Student's *t*‐test).
**Figure S2:** The number following “P” indicates the passage number. Whole‐cell lysates were prepared from HOK passaged as described. Cell lysates were immunoblotted with anti‐Lamin B1, anti‐p21^Waf1/Cip1^, and anti‐GAPDH antibodies. A representative blot of three independent experiments is shown in Figure 2F. The bar graph shows the integrated signal intensity ratios of Lamin B1/GAPDH and p21^Waf1/Cip1^/GAPDH and expressed as the mean ± SEM from triplicate assays. *p < 0.05 and **p < 0.01 versus P4 cells (Dunnett's multiple comparison test).
**Figure S3:** MSEC (P3) were treated with medium alone (Med), G3‐YSD Control (G3C), or G3‐YSD (G3) (1 μg/mL each) for 1 h. Cell lysates were immunoblotted with anti‐IκBα and anti‐GAPDH antibodies. A representative blot of two independent experiments is shown.
**Figure S4:** Detection of CK19 (a ductal cell marker), AQP5 (an acinar cell marker), and α‐amylase (a serous acinar cell marker) protein expression in A253 (A), IHSGEC (I), and MSEC (M: passaged three times). Whole‐cell lysates prepared from these cells were immunoblotted with anti‐CK19, anti‐AQP5, anti‐α‐amylase, and anti‐GAPDH antibodies. A representative blot of two independent experiments is shown.
**Figure S5:** HGF (P3) were treated with medium alone (Med), G3‐YSD Control (G3C), or G3‐YSD (G3) (2.5 or 10 μg/mL each) for 48 h. The concentration of IL‐1β in the medium was assayed by ELISA. Data represent the mean ± SEM of triplicate assays. ND, not detected.
**Figure S6:** The number following “P” indicates the passage number. Whole‐cell lysates were prepared from MSEC passaged as described. Cell lysates were immunoblotted with anti‐phospho‐(p)NF‐κB, anti‐NF‐κB, and anti‐GAPDH antibodies. A representative blot of three independent experiments is shown. The bar graph shows the integrated signal intensities of the pNF‐κB/NF‐κB ratio and the mean ± SEM of triplicate assays. **p < 0.01 versus P1 cells (Dunnett's multiple comparison test).
**Figure S7:** HOK (passaged three times) treated with indicated concentrations of *Pg* LPS, as described in the Materials and Methods, for 24 h. Whole‐cell lysates were immunoblotted with anti‐LCN2 and anti‐GAPDH antibodies. A representative blot of three independent experiments is shown. The bar graph shows the integrated signal intensities of the LCN2/GAPDH ratio and the mean ± SEM of triplicate assays. Not significant (NS) versus *Pg* LPS 0 μg/mL cells (Dunnett's multiple comparison test).
**Figure S8:** HOK (passaged three times) treated with indicated concentrations of IL‐1β, as described in the Materials and Methods, for 48 h. Whole‐cell lysates were immunoblotted with anti‐γH2A.X and anti‐GAPDH antibodies. A representative blot of two independent experiments is shown.
**Figure S9:** Whole‐cell lysates were prepared from HOK passaged as described. Cell lysates were immunoblotted with anti‐keratin 5, anti‐LCN2, and anti‐GAPDH antibodies. A representative blot of two independent experiments is shown.

## Data Availability

The data that support the findings of this study are available from the corresponding author upon reasonable request.
