# Supplementary material for: Impact of Cellular Senescence on LCN2 Expression in Salivary Gland Epithelial Cells and Oral Keratinocytes
Source: Biofactors. 2026 Feb 18;52(1):e70087. doi: 10.1002/biof.70087 (PMC12916262; doi:10.1002/biof.70087)
Supplement: Supplementary file 1 — Figure S1: The number following “P” indicates the passage number. Whole‐cell lysates were prepared from MSEC passaged as described. Cell lysates were immunoblotted with anti‐Perilipin 2 and anti‐GAPDH antibodies. The bar graph shows the integrated signal intensities of the Perilipin 2/GAPDH ratio and the mean ± SEM of triplicate assays. *p < 0.05 (the unpaired Student's t‐test). Figure S2: The number following “P” indicates the passage number. Whole‐cell lysates were prepared from HOK passaged as described. Cell lysates were immunoblotted with anti‐Lamin B1, anti‐p21Waf1/Cip1, and anti‐GAPDH antibodies. A representative blot of three independent experiments is shown in Figure 2F. The bar graph shows the integrated signal intensity ratios of Lamin B1/GAPDH and p21Waf1/Cip1/GAPDH and expressed as the mean ± SEM from triplicate assays. *p < 0.05 and **p < 0.01 versus P4 cells (Dunnett's multiple comparison test). Figure S3: MSEC (P3) were treated with medium alone (Med), G3‐YSD Control (G3C), or G3‐YSD (G3) (1 μg/mL each) for 1 h. Cell lysates were immunoblotted with anti‐IκBα and anti‐GAPDH antibodies. A representative blot of two independent experiments is shown. Figure S4: Detection of CK19 (a ductal cell marker), AQP5 (an acinar cell marker), and α‐amylase (a serous acinar cell marker) protein expression in A253 (A), IHSGEC (I), and MSEC (M: passaged three times). Whole‐cell lysates prepared from these cells were immunoblotted with anti‐CK19, anti‐AQP5, anti‐α‐amylase, and anti‐GAPDH antibodies. A representative blot of two independent experiments is shown. Figure S5: HGF (P3) were treated with medium alone (Med), G3‐YSD Control (G3C), or G3‐YSD (G3) (2.5 or 10 μg/mL each) for 48 h. The concentration of IL‐1β in the medium was assayed by ELISA. Data represent the mean ± SEM of triplicate assays. ND, not detected. Figure S6: The number following “P” indicates the passage number. Whole‐cell lysates were prepared from MSEC passaged as described. Cell lysates were immu [file BIOF-52-0-s001.docx]

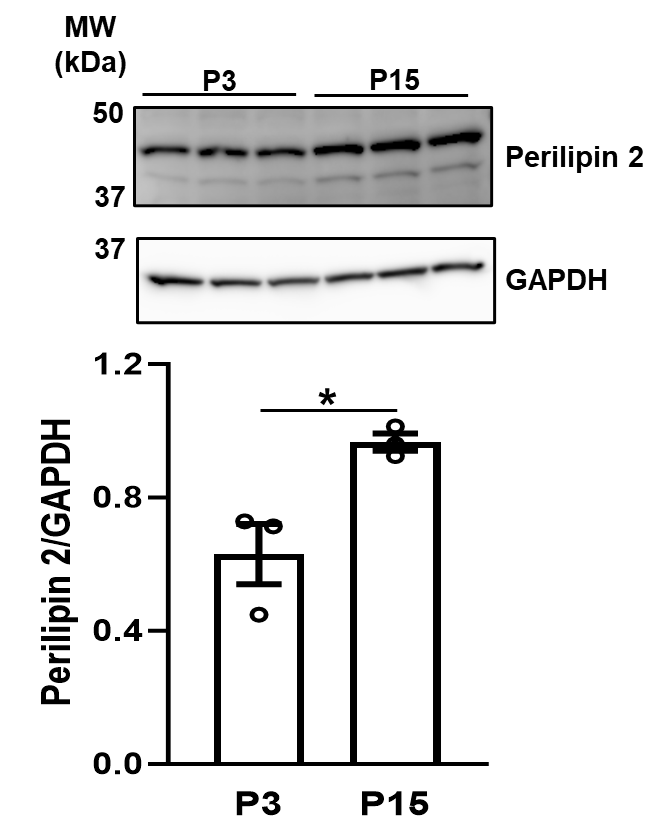


Supplementary Fig. 1. The number following ‘P’ indicates the passage number. Whole-cell lysates were prepared from MSEC passaged as described. Cell lysates were immunoblotted with anti-Perilipin 2 and anti-GAPDH antibodies. The bar graph shows the integrated signal intensities of the Perilipin 2 /GAPDH ratio and the mean ± SEM of triplicate assays. *P < 0.05 (the unpaired Student’s *t*-test).


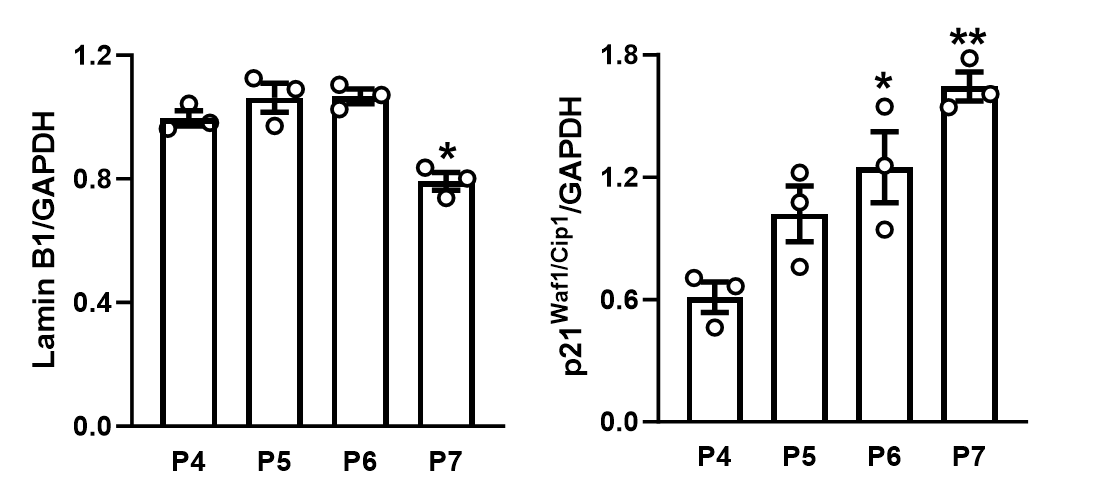


Supplementary Fig. 2. The number following ‘P’ indicates the passage number. Whole-cell lysates were prepared from HOK passaged as described. Cell lysates were immunoblotted with anti-Lamin B1, anti-p21^Waf1/Cip1^, and anti-GAPDH antibodies. A representative blot of three independent experiments is shown in Fig. 2F. The bar graph shows the integrated signal intensity ratios of Lamin B1/GAPDH and p21^Waf1/Cip1^/GAPDH and expressed as the mean ± SEM from triplicate assays. *P < 0.05 and **P < 0.01 versus P4 cells (Dunnett’s multiple comparison test).


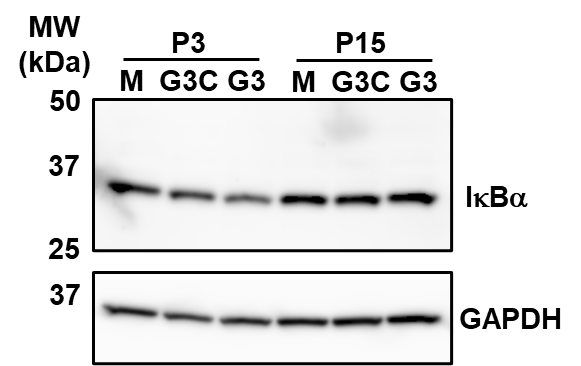


**Supplementary Fig. 3.** MSEC (P3) were treated with medium alone (Med), G3-YSD Control (G3C), or G3-YSD (G3) (1 μg/ml each) for 1 h. Cell lysates were immunoblotted with anti-IκBα and anti-GAPDH antibodies. A representative blot of two independent experiments is shown.


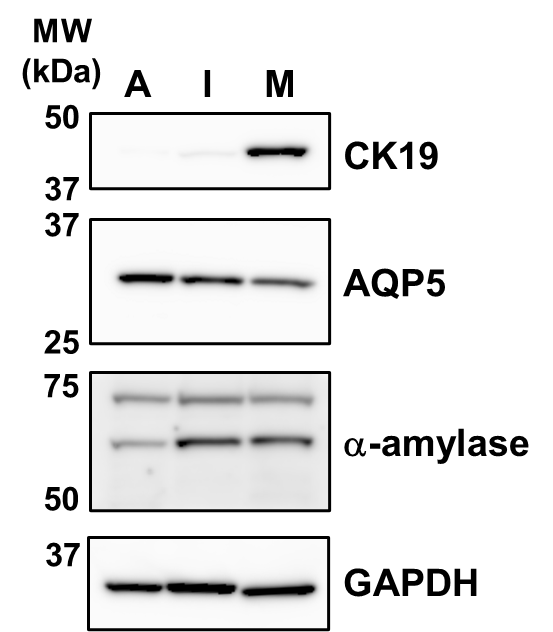


**Supplementary Fig. 4.** Detection of CK19 (a ductal cell marker), AQP5 (an acinar cell marker), and α-amylase (a serous acinar cell marker) protein expression in A253 (A), IHSGEC (I), and MSEC (M: passaged three times). Whole-cell lysates prepared from these cells were immunoblotted with anti-CK19, anti-AQP5, anti-α-amylase, and anti-GAPDH antibodies. A representative blot of two independent experiments is shown.


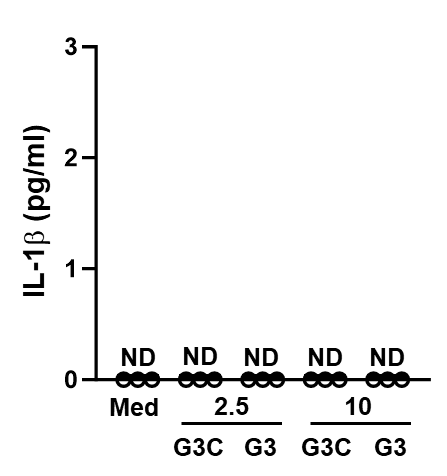


**Supplementary Fig. 5.** HGF (P3) were treated with medium alone (Med), G3-YSD Control (G3C), or G3-YSD (G3) (2.5 or 10 μg/ml each) for 48 h. The concentration of IL-1β in the medium was assayed by ELISA. Data represent the mean ± SEM of triplicate assays. ND: not detected.


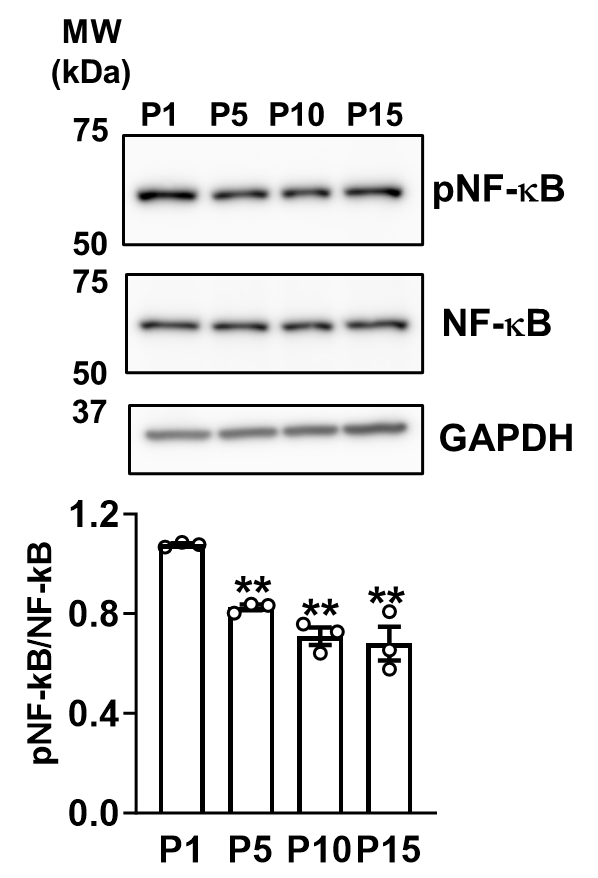


**Supplementary Fig. 6.** The number following ‘P’ indicates the passage number. Whole-cell lysates were prepared from MSEC passaged as described. Cell lysates were immunoblotted with anti-phospho-(p)NF-κB, anti-NF-κB, and anti-GAPDH antibodies. A representative blot of three independent experiments is shown. The bar graph shows the integrated signal intensities of the pNF-κB/NF-κB ratio and the mean ± SEM of triplicate assays. **P < 0.01 versus P1 cells (Dunnett’s multiple comparison test).


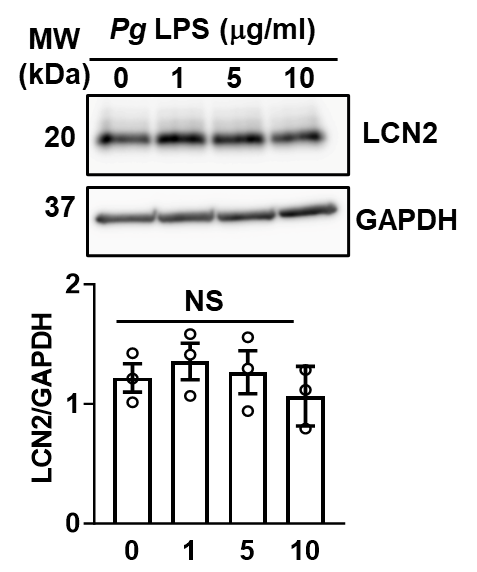


**Supplementary Fig. 7.** HOK (passaged three times) treated with indicated concentrations of *Pg* LPS, as described in the Materials and Methods, for 24 h. Whole-cell lysates were immunoblotted with anti-LCN2 and anti-GAPDH antibodies. A representative blot of three independent experiments is shown. The bar graph shows the integrated signal intensities of the LCN2/GAPDH ratio and the mean ± SEM of triplicate assays. Not significant (NS) versus *Pg* LPS 0 μg/ml cells (Dunnett’s multiple comparison test).


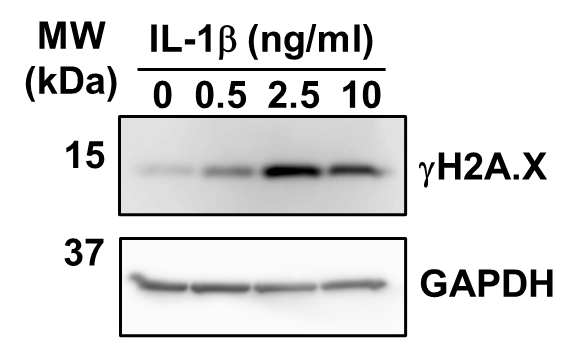


**Supplementary Fig. 8.** HOK (passaged three times) treated with indicated concentrations of IL-1β, as described in the Materials and Methods, for 48 h. Whole-cell lysates were immunoblotted with anti-γH2A.X and anti-GAPDH antibodies. A representative blot of two independent experiments is shown.


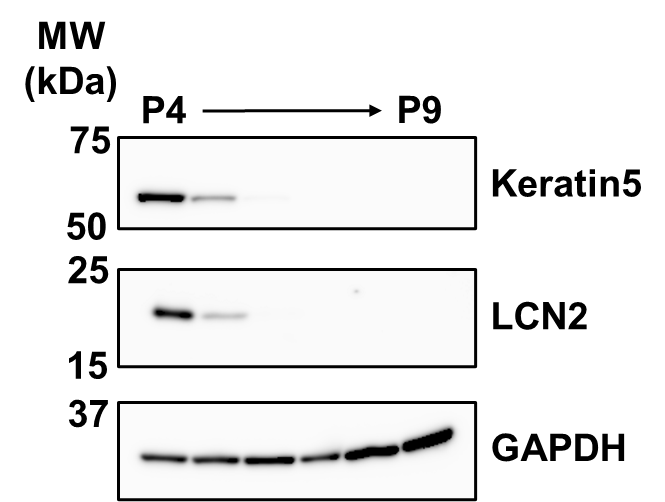


**Supplementary Fig. 9.** Whole-cell lysates were prepared from HOK passaged as described. Cell lysates were immunoblotted with anti-keratin 5, anti-LCN2, and anti-GAPDH antibodies. A representative blot of two independent experiments is shown.
